# Professor Von Bergmann

**Published:** 1907-04

**Authors:** 


### OBITUARY.

Professor von Bergmann, the famous surgeon, died at Wiesbaden, March 25, 1907. He was operated upon the day before for intestinal disorder without an anesthetic and bore the prolonged cutting with the greatest fortitude, although he did not direct the surgery, as was the case in a previous operation a few months ago. The dead surgeon was the greatest authority on gunshot wounds in Germany.

Ernst von Bergmann was born in the Russian Baltic province of Livonia, December 16, 1836. He studied medicine at the universities of Dorpat, Vienna and Berlin, and was graduated from the medical department of Dorpat in 1864. During the Austro-Prussian War of 1866 he was placed in charge of the military hospital at Königinhof, in Bohemia, and during the Franco-Prussian War he was at the head of the military hospitals of Mannheim and Carlsruhe. In 1875 he was appointed to the chair of surgery in the University of Dorpat, remaining there until the breaking out of the Turco-Russian War, when he became attached to the Russian Army of the Danube as consulting physician. Returning to Germany, Dr. von Bergmann was made surgeon in chief of the hospital at Würzburg and professor of surgery at the university. In 1882 he was called to the chair of surgery at the University of Berlin, to succeed Professor von Langenbeck, and also had charge of the surgical clinic of that city.

When, on December 16, 1906, Dr. von Bergmann attained the age of seventy the event was made the occasion of a notable celebration. No fewer than forty delegations called on him during the day to pay their respects. For five hours they filed past him, among them representatives of many universities, of the Red Cross, of various medical societies and other associations and of the German Samaritan Society. In recognition of his valuable services in war time and to education the ministers of War and Education were represented by government officials of high rank. Four important scientific works were published to commemorate the anniversary and Dr. von Bergmann's services to surgery. At a dinner in his honor in the evening more than five hundred prominent persons were present by invitation of the Berlin Medical Society.

Not only was Dr. von Bergmann a great surgeon and scientist, but his far reaching sympathies extended into many other fields. He was an ardent lover of art, of music and of the drama; while, as one writer has said of him, "he was recognised as one of the leaders among those who work for the public weal." He was a pioneer in antiseptic surgery, and made notable advances in investigation of diseases of the brain.

The professional activities of Dr. von Bergmann were continued up to a recent date. In May, 1906, he was called to Constantinople, in consultation, during the illness of the Sultan's daughter, Sultana Ayisheh; in July he presided at a special meeting of the Berlin Medical Association, called to discuss the treatment of appendicitis; in August he was once more summoned to Turkey, this time to attend the Sultan himself, and early in 1907 he treated the late Shah of Persia, for which he is said to have received a fee of $22,000. He was the author of numerous treatises on surgery, among them "Zur Lehre von der Fettembolie," "Die Resultate der Gelenkresektionen im Kriege," "Die Lehre von den Kopfverletzungen" and "Die Chirurgische Behandlung von Hirnkrankheiten."

